# Gastrointestinal Dysmotility in MNGIE: from thymidine phosphorylase enzyme deficiency to altered interstitial cells of Cajal

**DOI:** 10.1186/s13023-019-1016-6

**Published:** 2019-02-08

**Authors:** Rana Yadak, Marjolein Breur, Marianna Bugiani

**Affiliations:** 1 Department of Neurology, Erasmus University Medical Center, Rotterdam, The Netherlands; 2 Department of Child Neurology, VU University Medical center, Amsterdam, The Netherlands; 3 Department of Pathology, VU University Medical Center, Amsterdam, The Netherlands

**Keywords:** MNGIE, Mitochondrial neurogastrointestinal encephalomyopathy, Chronic intestinal pseudo-obstruction, CIPO, Interstitial cells of Cajal, ICC, HSCT, Intestinal organoids

## Abstract

**Background:**

MNGIE is a rare and fatal disease in which absence of the enzyme thymidine phosphorylase induces systemic accumulation of thymidine and deoxyuridine and secondary mitochondrial DNA alterations. Gastrointestinal (GI) symptoms are frequently reported in MNGIE patients, however, they are not resolved with the current treatment interventions.

Recently, our understanding of the GI pathology has increased, which rationalizes the pursuit of more targeted therapeutic strategies. In particular, interstitial cells of Cajal (ICC) play key roles in GI physiology and are involved in the pathogenesis of the GI dysmotility. However, understanding of the triggers of ICC deficits in MNGIE is lacking. Herein, we review the current knowledge about the pathology of GI dysmotility in MNGIE, discuss potential mechanisms in relation to ICC loss/dysfunction, remark on the limited contribution of the current treatments, and propose intervention strategies to overcome ICC deficits. Finally, we address the advances and new research avenues offered by organoids and tissue engineering technologies, and propose schemes to implement to further our understanding of the GI pathology and utility in regenerative and personalized medicine in MNGIE.

**Conclusion:**

Interstitial cells of Cajal play key roles in the physiology of the gastrointestinal motility. Evaluation of their status in the GI dysmotility related to MNGIE would be valuable for diagnosis of MNGIE. Understanding the underlying pathological and molecular mechanisms affecting ICC is an asset for the development of targeted prevention and treatment strategies for the GI dysmotility related to MNGIE.

## Background

Mitochondrial neurogastrointestinal encephalomyopathy (MNGIE) is a rare metabolic disease.

More than 120 MNGIE cases were reported [1]. MNGIE is caused by mutations in the nuclear gene *TYMP* [2], and is inherited in an autosomal recessive manner. A variety of pathogenic mutations in *TYMP* have been reported that are responsible for the detrimental lack of thymidine phosphorylase enzyme activity [3]. Lack of thymidine phosphorylase enzyme activity causes the systemic accumulation of the substrates pyrimidine deoxyribonucleosides, thymidine (dThd) and deoxyuridine (dUrd) [4], which disturbs deoxyribonucleoside triphosphates (dNTPs) pools [5]. Consequently, alterations in mitochondrial DNA (mtDNA) stability occur [6, 7].

Ethnic predisposition for MNGIE is not observed, however, certain *TYMP* mutations were reported prevalent in specific locations, for example, c.866A > G in Europe [8].

Clinical variability has been reported among MNGIE patients. For example, some patients present with mild clinical involvement of the gastrointestinal tract despite the presence of mutations in *TYMP* and marked reduction in TP activity [9]. Clinical variability also occurs between members of the same MNGIE family [10, 11]. Altogether, these data suggest that environmental factors (e.g. diet, life style, medicine history) might contribute to the manifestations of MNGIE. However, so far, no direct evidence has been reported in this regard. Furthermore, the shift of the gut microbiota might be involved in the manifestation or aggregation of the gastrointestinal (GI) dysmotility in MNGIE. Similar association has been addressed in other gastrointestinal motility disorders including inflammatory bowel disease [12], irritable bowel syndrome [13], and celiac disease [14]. Generally, MNGIE patients exhibit intestinal bacterial overgrowth [1]. The mitochondrial abnormalities observed in MNGIE perhaps contribute to this disturbed microbiota homeostasis. In this regard, one study shows that mitochondrial dysfunction (reflected by respiratory chain deficiency) detected in the colon of *PolgA*^*mut*^/^*mut*^ mice model of aging, is associated with changes in their gut microbiota homeostasis [15].

MNGIE is frequently associated with chronic intestinal pseudo-obstruction (CIPO), a syndrome of intestinal obstruction symptoms without the presence of an anatomical or mechanical obstruction, that eventually leads to severe gut motility failure [16]. Symptomatic management of CIPO includes the use of prokinetic agents to relieve dysmotility symptoms, and antinociception drugs or splanchnic nerve blockage to control abdominal pain [17]. The pathophysiology of CIPO involves inability of peristalsis and propulsion of intestinal contents as a result of disturbed neuro-muscular coordination due to myopathic (affects the intestinal contraction), neuropathic (affects the coordination of enteric reflexes) [16, 18], or mesenchymopathies related to abnormalities of the interstitial cells of Cajal (ICC) [19].

Allogenic hematopoietic stem cell transplantation (HSCT), is currently the available treatment for MNGIE [20]. In most cases, CIPO- related malnutrition persists hence parenteral nutrition is required [21]. Gastrointestinal complications are the main mortality factor in MNGIE patients and the least treatable with the currently available therapies. The limited benefits of the current treatments aiming to relieve the GI symptoms relate to the inadequate understanding of the molecular mechanisms underlining the GI dysmotility in MNGIE. In this article, we provide an overview of the current knowledge of the GI dysmotility in MNGIE, with a particular focus on ICC due to their central physiological role in GI motor activity, and the growing evidence supporting their role in etiology of GI dysmotility in multiple pathologies [22]. We summarize the current knowledge about ICC development, function, and roles in GI dysmotility, and discuss molecular mechanisms in which multiple factors probably attribute to development of ICC abnormalities. Finally, we discuss the currently available treatments, potential future prevention and therapeutic strategies, to address the GI dysmotility in MNGIE patients.

### Pathological aspects of GI dysmotility in MNGIE

Most of the knowledge about the GI dysmotility in MNGIE is inferred from the pathological evaluation of intestinal tissues. MNGIE patients often reveal visceral myopathy, mainly atrophy and fibrosis of the external layer of the muscularis propria of the small intestine [23–28], and neurogenic changes that involve the myenteric plexus and ganglion cells [24–26, 29] (Table 1). Changes in mitochondrial morphology were also reported, including abnormally shaped and large mitochondria in the smooth muscle cells (SMCs) of the small intestine, and ganglion cells of the entire GI tract [24, 27, 29]. Molecular investigation revealed mtDNA depletion in five MNGIE patients, selectively in the muscularis propria external layer of the small intestine, which also displayed atrophy and fibrosis, establishing a link between abnormal mitochondrial DNA genetics and visceral myopathy [30, 28]. Additional to visceral myogenic and neurogenic changes, ICC and ICC networks were reported absent in MNGIE [31], and due to their vital roles in GI physiology, they represent valuable targets for prevention and treatment of GI dysmotility as discussed below.

**Table 1:** Summary of microscopic features in the gut of MNGIE cases

Study	Gender, age (years)/ total body weight (kg)	Myogenic/ Neurogenic Histopathology	Remarks
Bardosi A, et al. [23]	Female, 42, 40	Fibrosis of SI submucosa and subserosa, hypertrophy of the tunica MM	–
Perez-Atayde AR, et al. [24, 25]	-Female, 14, − -Male, 14, −	Atrophy and fibrosis of the smooth muscle EL of MP, residual smooth muscle myocytes show cytoplasmic vacuoles, cytoplasmic eosinophilic inclusions (megamitochondria) observed by light microscopy in smooth muscle myocytes of MM and MP of the esophagus and SI and ganglion cells of the entire GI	These studies suggest that noninvasive rectal biopsy can contribute to the diagnosis of MNGIE in additional to the standard diagnostic criteria [108]. Abnormal intestinal mitochondrial morphology/ genetics/ function as diagnostic markers for MNGIE.
Teitelbaum JE, et al. [29]	-Female, 14, 23	Focal muscle absence, serosal granulomas, fibrosis, megamitochondria in SMCs of the MM (rectal suction biopsy), focal loss of Auerbach’s plexus, megamitochondria in ganglion cells of the MM (rectal suction biopsy)	–
Szigeti K, et al. [26]	-Male, 17, −	Atrophy of longitudinal EL of MP, hypertrophy of the inner circular smooth muscle bundles of the MP, swollen SMCs with pale cytoplasm, enlarged nerve cells, ganglion cells were infrequent	–
Blondon H, et al. [27]	-Female, 26, 39 -Female, 30, 28 -Male, 22, 43	Atrophy and fibrosis of the EL of MP and vacuolated SMCs, hypertrophy of the inner layer of the MP, bundance of abnormally shaped megamitochondria with lipid droplets (EM) in MP smooth muscle myocytes (SI and gallbladder)	–
Giordano C, et al. [28, 30]	5 cases, summarized in reference [18]	Chronic inflammation (mucosa), edema (submucosa), preserved inner layer of MP, atrophy and interstitial fibrosis limited to the smooth SMCs of the longitudinal EL of MP (stomach and more pronounced in SI), pyconic nuclei and cytoplasmic microvaculation in the smooth muscle cells of the EL (SI), vacuolated cytoplasm filled with swollen mitochondria and lipids (EM)	- Low levels of mtDNA point mutations in myocytes, nerve fibers and ganglia of the myenteric plexus. - No mtDNA deletions in SI. - Mitochondrial proliferation and mtDNA depletion limited to the EL of the MP of the entire GI. - mtDNA depletion correlates with the atrophy and interstitial fibrosis - Baseline mtDNA levels are low in MP smooth muscles of SI of a normal subject, predisposing the SI smooth muscles to nucleoside imbalances and selective SI myopathy in MNGIE patients.
Zimmer V, et al. [31]	- Female, 35, 31	Absence of the normally abundant c-Kit-positive ICC around the myenteric plexus, in intermuscular septa and within muscular plexus	- Absence of ICC can be an early event of GI dysmotility preceding myo/neurogenic morphological changes. - Absence of ICC can be due to cell death, transdifferentiation into smooth muscle phenotype or their loss can be a secondary event due to mitochondrial energy failure. - Dysfunctional ICC networks account for disturbed pacemaker activity and interfere with neurotransmission.
Yadak R, et al. [71]	3 cases summarized in reference [61]	Fibrosis and atrophy of the external layer of the tunica muscularis propria, ICCs were completely lost in all MNGIE patients	Intestinal muscle wall atrophy and complete loss of Cajal cells in treated patients were not recovered after HSCT.

*EL* external layer, *EM* electron microscopy, *GI* gastrointestinal, *HSCT* hematopoietic stem cell transplantation, *ICC* interstitial cells of Cajal, *MM* muscularis mucosae, *MP* muscularis propria, *SI* small intestine, *SMCs* small muscle cells

## Interstitial cells of Cajal (ICC)

### Development and function

ICC and longitudinal smooth muscle cells share a common embryonic origin [32, 33]. During embryonic development, the mesenchymal progenitors express the receptor tyrosine kinase c-Kit and smooth muscle myosin heavy chain [34]. Upon stimulation by stem cell factor (SCF), these precursors would normally turn into interstitial cells in the myenteric region (ICC_MY_), otherwise, they develop into the longitudinal smooth muscle layers of the mammalian small intestine [35]. Blocking c-Kit signaling hinders the development of the ICC network, probably by transdifferentiation of ICC_MY_ into a smooth muscle cell-like phenotype [36].

SCF/c-Kit signaling pathway is also important to maintain the function of ICC. This was demonstrated by disturbance of normal GI motility through selective loss of ICC with cessation of the slow waves and significant reduction of neurotransmission in the mouse bowels upon blockage of the c-Kit receptor by neutralizing Kit antibodies [37, 38]. Indeed, a strong in vivo evidence for the role of SCF/c-Kit signaling for development of ICC networks, and the physiological role of ICC as pacemakers of the gut, is provided by generation of the *kit* and steel factor (c-Kit ligand) mutant mice. Blocking of the SCF/c-Kit signaling in *kit* mutant mice diminished the number of ICC_MY_ in the Auerbach’s myenteric plexus and the slow waves and propulsive contractile activity [39, 40]. Signs of impaired growth rate are reported in adult steel factor mutant mice, probably related to attenuated segmentation motility necessary for absorption of nutrients [41]. In addition to their contribution to peristalsis which facilitates propulsion of intestinal contents, and small intestine segmentation which facilitates absorption of nutrients [32], ICC play a role in mediating motor neurotransmission between smooth muscles and motor neurons [42].

### Anatomical location and markers

**Fig. 1:**
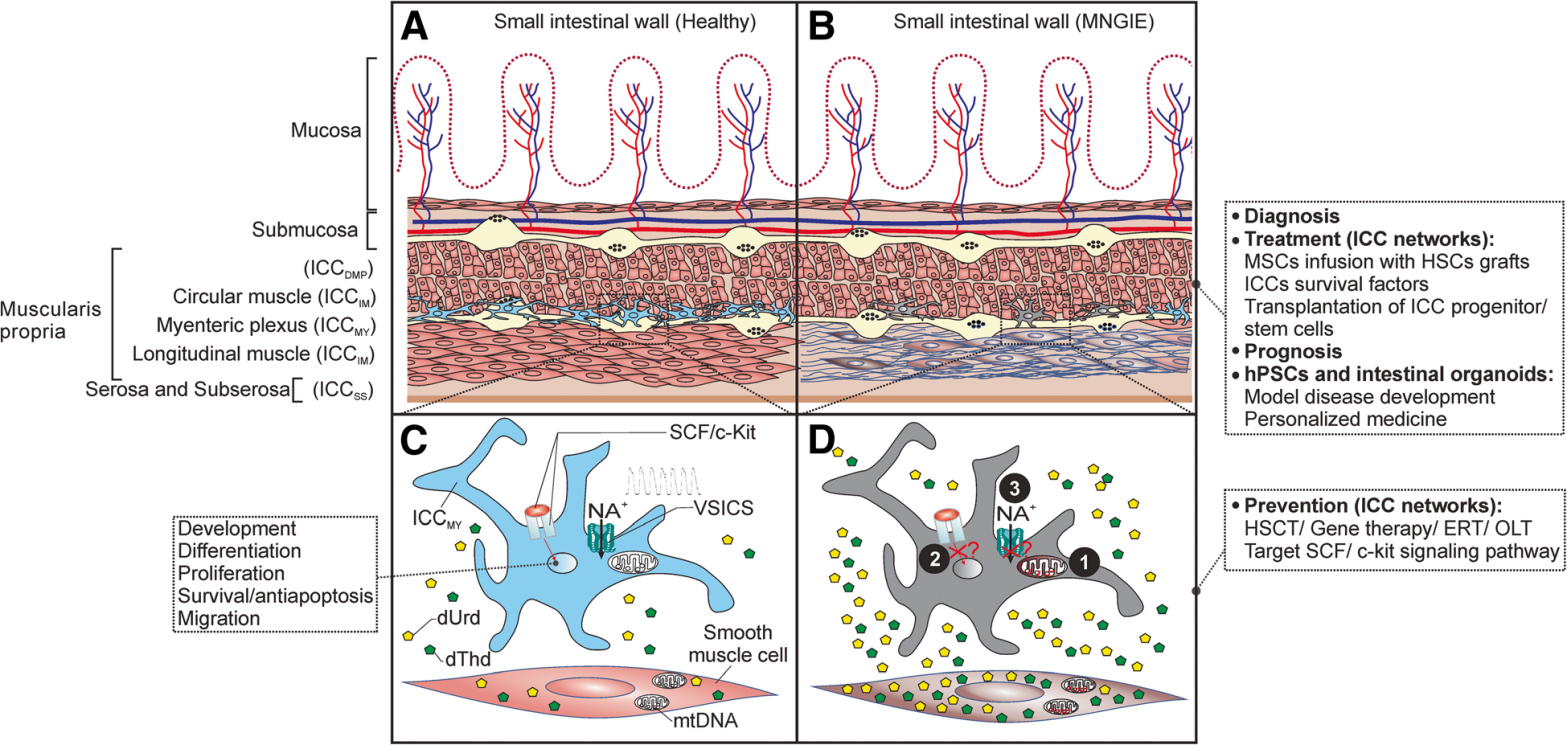
Pathogenesis of altered ICC networks in MNGIE, prevention and treatment prospects. **a** Representation of the anatomical localization of the subtypes of interstitial cells of Cajal (ICC) in the human small intestine (SI). The specific types of ICC are indicated in parenthesis. Depicted are the ICC_MY_ (blue), prominent and associated with the ganglia of the myenteric plexus region. ICC_MY_ are the primary pacemaker cells in the small intestine, responsible for generation and propagation of electrical slow waves and are rich with mitochondria [59]. **b** ICC/ ICC networks are compromised in MNGIE. ICC/ ICC networks are disturbed and ICC_MY_ are depleted in MNGIE patients. Dysfunction of ICC networks is thought to precede the myo/ neurogenic morphological changes [31], mainly atrophy and fibrosis of the longitudinal muscle external layer of muscularis propria (depicted). Additionally to typical symptoms and biochemical parameters, at this stage, gastrointestinal biopsies could serve to confirm diagnose of MNGIE as well as to evaluate the status of ICC networks prior to treatment to predict the therapeutic outcome on GI manifestations and monitor the impact of treatment on the restoration of ICC networks (prognosis). This is made feasible by routine histology of the gastrointestinal biopsies [24]. **c, d** Proposed molecular mechanisms responsible for impaired ICC networks in MNGIE. **c** SCF/c-Kit signaling pathway is necessary for development, proliferation, maintenance of ICC/ ICC function, and voltage-sensitive ion channels (VSICs) are necessary for generation and propagation of electrical slow waves and motor activity of ICC. **d** In MNGIE, however, loss of ICC/ICC networks and dysfunctional ICC might be a secondary event due to altered thymidine (dThd) and deoxyuridine (dUrd) levels that leads to **(1)** mitochondrial DNA (mtDNA) instability, mitochondrial energy failure, interference with mitochondrial-mediated Ca^2+^ cycling [61] and cells death (mtDNA depletion is also reported in the smooth muscle cells of the longitudinal muscle external layer of muscularis propria [28]). In addition, unbalanced nucleosides might attribute to **(2)** Blocking of SCF/c-Kit signaling, which hinders the development, proliferation and maintenance of function of ICC networks, probably by transdifferentiation of ICC_MY_ into a smooth muscle cell-like phenotype or cell death [36], and probably to **(3)** Disturbance of the VSICs such as Ano1 or Na_v_1.5, which impairs generation and propagation of electrical slow waves, SI contractions and motility [64], or **(4)** the homing capacity of the BM-derived ICC to the small intestine might be compromised **(not shown)**. Multiple mechanisms might act together. **Prevention interventions** are applied to cease further deterioration of the ICC/ ICC networks, therefore preferably applied at earlier stages of the disease progression. **Treatment interventions** focus on restoration of damaged ICC networks. Infusion of MSCs along with HSCT not only promotes homing, survival and quiescence of HSCs but also could replenish the ICC pool in the small intestine by differentiation into ICC and homing to SI. Novel technologies to improve the homing capacity of MSCs and promote their engraftment in the SI could involve enhanced ex vivo cell culture conditions, gene modifications or coating with antibodies into cell surface. Additionally, administration of candidate survival factors of residual ICC stem/ progenitor cells would replenish remaining ICC pools [76, 77]. ICC stem/ progenitor cells can be selected via cell surface markers for transplantation, alternatively, human pluripotent stem cells (hPSCs) would advance our knowledge about- and provide an accessible source of ICC. Healthy and MNGIE patients- derived organoids are expected to accurately model GI dysmotility development and prevention and eventually be applied clinically in personalized and regenerative medicine

Interstitial Cajal-like cells are located in multiple organs outside the GI tract, including the pancreas, placenta, and the female reproductive tract [43]. In the GI tract, ICC are localized at different levels including the esophagus, stomach, pancreas and large intestine [44]. In the small intestine, ICC are associated with the two nerve plexuses, mainly within the intermuscular space between the two muscle layers in the Auerbach’s myenteric plexus (ICC_MY_) or within the deep muscular plexus region between the circular thin and thick muscle layers (ICC_DMP_). ICC _DMP_ occur only in the small intestine [45] **(**Fig. 1). While ICC_MY_ generate and propagate electric rhythmicity, ICC_DMP_ are associated with the nerve bundles of the deep muscular plexus and mediate neuronal inputs [46]. Intramuscular ICC in the circular or the longitudinal layers (ICC_IM_) are also found in other parts of the GI tract and mediate motor neuronal input. Subserosal ICC (ICC_SS_) are found in the small intestine and colon. ICC around the submucosa of the pylorus and colon (ICC_SM_) are involved in pacemaker activity and neuronal input [35]. In addition to the anatomical location, ICC are grouped based on their morphology and primary function [47].

The property that ICC express c-Kit receptor and the fact that SCF/c-Kit signaling cascades are important for development and function of ICC as demonstrated in c-Kit or SCF mutant mouse models [35], accelerated the understanding of the (patho-)physiology of these cells. In addition to morphological and ultrastructural characterization, c- Kit receptor (CD117) has been widely recognized as a selective marker for detection of ICC by immunohistochemistry in human clinical samples, and elucidation of their properties and interaction with SMCs and neurons [48, 49]. A few co-transporters and receptors selectively expressed on ICC and Ano1 chloride channels were reported as additional specific markers for detection of ICC [44]. The phenotype Kit^low^CD44 + CD34 + Insr+Igf1r + identifies a population of ICC progenitors/ stem cells in murine gastric muscles [50].

### Involvement in GI motility disorders

Quantitative and qualitative abnormalities of ICC/ ICC networks are implicated in CIPO. c-kit+ ICC were reported absent in the intestine of two patients with the myopathic form of CIPO [51], in the small and large intestine of six cases of idiopathic CIPO [48], and in the distal ileum and colon of a pediatric case of intestinal pseudoobstruction [49]. Abnormal distribution of ICC/ ICC networks was reported in the small intestine and colon of pediatric and adult patients with intestinal pseudoobstruction [52–54].

Beyond CIPO, altered numbers / depletion, distribution, morphology or ultrastructural changes were reported to occur in ICC/ ICC networks located in various parts of the gut in several chronic gastrointestinal motility disorders, including the esophagus (achalasia and gastroesophageal reflux), stomach (diabetic gastroenteropathy and infantile hypertrophic pyloric stenosis) and in patients with genetic alterations in *kit*; in the small intestine and colon (Hirschsprung’s disease, idiopathic megacolon, juvenile intestinal pseudo-obstruction, anorectal malformations, slow transit constipation) and in inflammatory bowel diseases (ulcerative colitis and Crohn’s disease) [35, 55, 56].

## Potential pathogenetic mechanisms of altered ICC networks in MNGIE

Due to their central roles as pacemakers of the gut and mediators of neurotransmission, alterations of ICC are strongly associated with GI motility disorders and are fundamental in the development and underlying pathogenesis of these disorders [19, 57]. Consequently, clarification of the causes of reduced / disturbed ICC networks in MNGIE provides research avenues to overcoming MNGIE enteropathy. The dynamics of disturbed ICC pools in disease might be caused by [58]: increased loss of ICC due to transdifferentiation or apoptosis, and / or reduced ICC pools due to attenuated development, proliferation and survival of stem cells, progenitors or mature ICC. Probably disease-specific environment/factors stimulate the observed quantitative and qualitative changes in ICC. For example, in MNGIE, the systemic accumulation of d-Thd and d-Urd nucleosides could contribute to either molecular mechanism by negatively affecting the following. ***i)*** **The mitochondria.** Essentially, the systemic accumulation of nucleosides most likely debilitates the ICC_MY_ of human intestine and stomach, which are particularly abundant in mitochondria [59]. This probably occurs by causing energy failure or disturbing the metabolic activity responsible for the propagation of the slow waves through mitochondria Ca^2+^ cycling [60], an important role of mitochondria in ICC [61]. This is in line with the findings that mtDNA depletion also occurs in the small intestine of MNGIE patients, leading to the assumption that this is a primary pathological event responsible for the GI dysmotility in MNGIE [28].

***ii)*** **The SCF/c-Kit signaling**. Imbalanced nucleosides pools could negatively influence the SCF/c-Kit signaling, vital for development and maintenance of ICC, thereby demolishing the ICC networks and pacemaker activity and the segmentation motility. For example, interruption of insulin/IGF-I signaling disturbs SCF/c-Kit signaling, reducing SCF levels which was implicated in loss of ICC in diabetic mice models [62, 63]. Additionally, blockage of SCF/c-Kit interactions leads to transdifferentiation of ICC to smooth muscle-like phenotypes [36]. Furthermore, attenuated SCF/c-Kit interactions could impair vital properties of stem cells, progenitors, or mature ICC impacting on proliferation, development and differentiation (Fig. 1). ***iii)*** **Voltage-sensitive ion channels (VSICs).** VSICs contribute to vital functions such as GI secretions, absorption and importantly, in motility as regulators of electro-mechanical activity of ICC and SMCs leading to contractions [64]. Targeting of VSICs has been suggested as a therapeutic intervention method for GI motility disorders associated with channelopathies, including irritable bowel syndrome [64]. Advantages of this approach would be the direct targeting of the final affected cells responsible for the pathology thereby eliminating potential side effects, and the vicinity of the effector cells to the lumen which facilitates drug delivery [65]. Evidence suggests that some calcium-inhibited and calcium-activated ion channels and sodium channels are involved in ICC functions, yet the pacemaker ion channel that initiates the slow wave is still not defined [65].

Near- complete knockout of ANO1, a calcium-activated- chloride channel expressed particularly by ICC, led to the loss of slow waves of the mouse small intestine [66]. Additionally, the voltage-gated sodium-selective ion channel (Na_V_1.5) contributes to the generation and propagation of the slow waves. Altered Na^+^ levels directly affect ICC as it results instantly in the disappearance of the ICC- generated electrical slow waves, supporting the vital role of Na^+^ current in generation and propagation of the electrical activity, the pacemaker property of ICC [67]. VSICs and ion currents might be impaired in MNGIE due to altered nucleosides, therefore responsible for the reduction in slow waves.

***iv)*** The intrinsic quality of the bone marrow-derived ICC might be compromised in MNGIE, reducing the capacity for homing to the small intestine, as previously reported in diabetic mice [68]. Although the aforementioned factors ***(i-iv)*** have not been tested in the context of MNGIE, they can be envisioned as plausible molecular mechanisms that, singularly or combined, might contribute to the overall declined quality, functionality and quantity of ICC/ ICC networks (Fig. 1). The status of the ICCs and networks and the above mentioned mechanisms could be explored in the Tymp^*−/−*^ Upp1^*−/−*^ mice [69], which model the systemic accumulation of d-Thd and d-Urd nucleosides [70]. In particular, it is of interest to evaluate whether ICC develop at all by studying embryonic or neonate Tymp^*−/−*^ Upp1^*−/−*^ mice [71], and the possibilities to prevent (further) loss of ICCs.

## Future prospects

### Additional diagnostic markers of MNGIE

Due to gastrointestinal symptoms similarity with other diseases, MNGIE has often been misdiagnosed as anorexia nervosa, inflammatory/ irritable bowel or celiac diseases [25]. This has led to a late diagnosis of MNGIE and patients received wrong treatments [29]. Therefore, early diagnosis can direct towards suitable treatments and early genetic consultation. Therefore, early diagnosis can direct towards suitable treatments and early genetic consultation.

The diagnosis of MNGIE is based on the evaluation of the patient history, clinical symptoms, magnetic resonance imaging of the brain, biochemical assessment, metabolic abnormalities such as in mitochondrial respiratory chain enzymes, mtDNA analysis, and genomic DNA screening for mutations in *TYMP* (reviewed in [3]). In particular, biochemical assessment is cardinal for the diagnosis of MNGIE and is important for the distinction between MNGIE and other GI motility disorders due to the typical findings in MNGIE patients [72]. In addition to these criteria, particularly biochemical testing and brain MRI, pathological diagnosis of GI dysmotility can be confirmative of MNGIE (Fig. 1).

In this regard, GI symptoms are cardinal in MNGIE, therefore diagnosis by GI biopsies can be more reliable than skeletal muscle biopsies which may show inconsistent pathological changes [26]. Evaluation of non- invasive rectal tissue by routine histology would provide a valuable diagnostic tool which shows megamitochondria readily observed as cytoplasmic eosinophilic inclusions by light microscopy [25]. On the other hand, the strong correlation between Kit immunoreactivity and ICC facilitated diagnosis of human GI motility disorders, and due to their role, ICC are suggested to be included as a diagnostic criterion for patients with symptoms of pseudo-obstruction [55]. Immunohistochemistry detection of c-kit+ ICC has been applied for this purpose. However it is important to support the IHC findings with morphology by routine and electron microscopy, other more recent molecular markers of ICC and perhaps a functional evaluation of ICC, preferably under standard criteria (IHC protocols, ICC counting methods). Based on histopathological criteria, The London classification 2010 provided guidelines for pathology standardized diagnosis of adult and pediatric gastrointestinal neuromuscular pathology [73], which would allow for proper diagnosis and treatment.

### Prevention and treatment strategies

In order to resolve the GI dysmotility in MNGIE, either (or both) molecular processes of increased loss/ reduced pools of ICC shall be targeted by preventive and therapeutic approaches.

Amelioration of altered TP/nucleosides metabolism by HSCT [20], gene therapy [70], orthotopic liver transplantation [74], or enzyme replacement therapy [75] (reviewed in [3]), singular or in combination with strategies focusing on overcoming the deficits of SCF/c-Kit pathways or VSICs, could be beneficial as preventive measures to maintain the quality and quantity of- and prevent further damage of intestinal ICC/ ICC networks (Fig. 1).

On the other hand, therapeutic approaches are applied at later stages of the disease and shall aim to replenish the stem cell/ progenitors or mature ICC pools and restore ICC networks. This includes cell therapies or administration of candidate survival factors that direct differentiation of residual ICC stem/ progenitor cells towards mature ICC and promote their proliferation (Fig. 1), such as SCF, neuronal nitric oxide [76], and exogenous serotonin [77]. In the following sections, we focus on cell therapy schemes as candidates to target deficits of ICC, and the avenues offered by the organoid and tissue engineering technologies to understand the pathology of GI dysmotility and ultimately their application in personalized medicine for MNGIE.

#### Cell therapies

##### Cell therapies in *kit* deficient mice

Cell therapy has been explored successfully under conditions that resemble pathological absence of ICC/ disturbed networks in wild-type and *kit* deficient mice. Firstly, cellular transplantation of ICC into the small intestine myenteric plexus of *kit* deficient mice restored the kit+ ICC_MY_ networks and pacemaker activity [78]. Technically, this allotransplantation approach is feasible due to ICC capacity to undergo mitotic division, however, it might require transplantation of full-thickness muscle strips from other parts of the GI or from a matched donor, being thus currently not clinically feasible in patients [79]. Secondly, the potential of bone marrow-derived mesenchymal stem cells (MSCs) to differentiate into ICC and repopulate injured ICC networks in the murine small intestine is established [79]. Following bone marrow transplantation (BMT), bone marrow-derived-ICC clusters were restored in the myenteric plexus of the irradiation injured small intestine of wild-type C57BL/6 mice [68, 80] and *kit* deficient mice, which normally lack ICC_MY_ networks and pacemaker activity [81, 82]. This demonstrated that BM-derived kit+ cells are capable to migrate to and repopulate the ICC_MY_ networks, although with conflicting outcomes on the recovery of motor activity in these studies, requiring further functional assessments [79].

##### Allogenic HSCT

Allogenic HSCT is currently the standard treatment for MNGIE [20] and has been proposed to be performed at early ages prior to GI manifestations in order to improve the therapeutic outcomes [20]. Concerning the GI pathology, our evaluation of GI tissues from MNGIE patients who received HSCT, demonstrate the presence of muscle wall atrophy and absence of ICC [71] (Table 1). On one hand, the study was limited by the small patient number and relatively short follow up time; on the other hand, the status of the ICC/ networks in MNGIE patients prior to transplantation and the potential contribution of BM-derived MSCs (see above) to recovery of ICC networks and GI motility has not been evaluated. In fact, GI manifestations are not consistently improved following HSCT [20], perhaps due to the severity of the damage occurred in ICC/ networks during the course of disease prior to treatment. Theoretically, this should imply ***i)*** an improved GI therapeutic outcomes in MNGIE patients who lack ICC or display disturbed ICC networks and ***ii)*** potential benefits of evaluation of the ICC/ networks in MNGIE patients prior to HSCT (diagnosis), which is made feasible by routine pathology examinations of biopsies, thereby ***iii)*** contribution to estimate the improvements of the GI motility (prognosis).

##### MSCs-based cell therapy

MSCs are multipotent stem cells capable to differentiate into multiple lineages and regulate core functions of HSCs such a migration, survival and support of hematopoiesis. MSCs are extensively being tested for their application in HSCT to enhance engraftment of transplanted HSCs and prevention of graft-versus-host disease [83]. Furthermore, MSCs are able to home to the injured tissues following total body irradiation when infused together with HSCs grafts, particularly home to the gastrointestinal organs, including the colon and small intestines of primates [84]. The ability to migrate into the injured sites and support tissue regeneration, as well as the immunomodulatory properties, render intestinal diseases good targets for treatment by MSCs cell-based therapy [85]. In MNGIE, additional to the above-mentioned contributions, BM-derived MSCs under the correct conditions could assist in the recovery of the lost or disrupted ICC/ networks. MSCs (usually isolated from bone marrow) could be infused in parallel with HSCs grafts as applied in clinical trials for hematological and solid malignancies [83].

Strategies to improve cellular homing and engraftment of the infused MSCs into the injured intestine can be inferred from bone marrow transplantation [86], myocardial infarction [87] and inflammatory bowel disease [88]. These may include ***(i)*** modification of the bio-distribution after systematic infusion of MSCs; ***(ii)*** adaptation of cell culture conditions to maintain the stem cell properties and enhance homing capacity (selected growth factors, chemokines or oxygen levels); and ***(iii)*** modulation of chemokine receptors and cell surface adhesion molecules (pre-treatment with growth factors, cytokine, genetic modifications of expression or coating with antibodies into cell surface).

##### ICC progenitors/ stem cells and human pluripotent stem cells

Murine ICC progenitors/ stem cells are identified by their Kit^low^CD44 + CD34 + Insr+Igf1r + (Kit^low^CD44 + CD34+) phenotype [50, 89] and contribute to the regeneration of ICC networks, however these primitive cells are rare. Research is required to identify such primitive cell populations in human, for selection and enrichment that, combined with ex vivo expansion technology, could be investigated for the feasibility of clinical application.

Neuronal progenitors and human induced pluripotent stem cells (hIPSCs) were demonstrated to generate specific GI neuronal cell types (reviewed in [90]), indicating the feasibility to generate human ICC under relevant differentiation conditions. HIPSCs-derived ICC would advance our knowledge by establishing parameters such as identification of specific markers of ICC, their signaling pathways, the pacemaker ion channels and regulators of the contractile activity, and involvement of ICC in pathogenesis [90].

#### Organoid technology and tissue engineering

Still in its infancy in the field of GI dysmotility, adult stem cells and hIPSCs are new tools both for basic and translational research. The in vitro grown human intestinal models are expected to advance our understanding of the molecular mechanisms of intestinal diseases with great potential for translational applications. The mini intestines are multi-cellular constructs produced in three dimensional (3D) cultures. Human intestinal epithelial organoids [91], generated from the primary small intestine (enteroids) or colon (colonoids) epithelial crypts adult stem cells (reviewed in [92]) and composed of all epithelial cells types. Human intestinal organoids (HIOs) maybe generated from hPSCs (embryonic stem cells (ESCs) or induced pluripotent stem cells (IPSCs) [93–95]. HIOs contain both epithelial and mesenchymal layers and can be developed into a system to mimic, to some degree, the cell composition, structure, physiology, and function of the intestine [95]. For a detailed review on organoids and their applications see [96–98]. The complexity of HIOs has been increased recently by tissue- engineering approaches when a functional enteric nervous system was incorporated into HIOs. These structures had functional plexi and ICC and exhibit neuronal contractile activity, representing a system to model the cellular and molecular basis of GI dysmotility in disorders like Hirschsprung’s disease [99]. Other tissue engineering developments illustrate the feasibility to perform autologous small intestine transplantation of tissue-engineered small intestines, for instance, when HIOs are supported with tubular shaped polymer scaffolds to support their growth in vivo [100]. This approach is being studied for the treatment of short bowel syndrome, with limitations to be addressed prior application in regenerative medicine [100, 101].

Intestinal organoids are generated from few starting materials, are capable to expand indefinitely, self-renew and remain stable for long periods under xenogeneic- free culture conditions [102] and are stably transduced by lentiviral vectors [103]. These properties would enable their utility as models for disease profiling, drug screening, designing personalized therapies and supply functional tissue for regenerative medicine [98], in particular for monogenic disorders. GI tissues of MNGIE patients are scarcely available, and a relevant model of MNGIE human intestine that accurately simulates the pathophysiology is lacking. The two-dimensional cell cultures [5, 7, 104] inadequately exhibit cellular function such as in tissues or modulate the disease-specific microenvironment. Notably, the available mouse model of MNGIE [69], although closely recapitulating the biochemical imbalances, does not display the GI manifestations; the fundamental physiological differences between the mouse and human would restrict translation to MNGIE patients [105].

On the other hand, healthy and MNGIE patients- derived intestinal epithelial organoids (from gut biopsy or surgically resected tissue stem cells) or –intestinal organoids (from skin-derived IPSCs or ESCs) would provide more physiologically relevant and tractable alternatives for the following potential applications [98]. ***i)*** **Disease characterization and identification of novel therapeutic targets**: MNGIE patients- derived epithelial/intestinal organoids would model the disease to study the morphological, structural and physiological changes, the status of the ICC networks, contractile activity, neuronal, neuro- muscular interactions, mtDNA alterations, and altered signaling pathways (by omics profiling). This could allow identification of novel molecules and pathways as therapeutic targets. ***ii)*** **Understanding disease development and identification of prevention strategies**: manipulation of healthy epithelial/intestinal organoids culture conditions to mimic intestinal biochemistry of MNGIE or gene editing of *TYMP*, would assist in evaluating the impact of microenvironment on early stages of GI dysmotility development, such as the contribution of deficit ICC networks, mtDNA alterations, principle signaling pathways, and their cross-talks. Therefore, this system would represent a platform to explore key prevention points during disease development. ***iii)*** **Personalized and regenerative medicine:** the ultimate goal of the organoids technology would be the treatment of the GI manifestations in MNGIE. MNGIE patient-derived intestinal organoids could be used to check the functional status (diagnosis), predict the potency and potential toxicity [106] of experimental platforms in resolving GI complications, and how well they would respond to treatment (prognosis). Intestinal organoids could be expanded for tissue regeneration or genetically modified by CRISPR/Cas9 [107] or vector [103] based gene therapy to restore TP, and perhaps supported by tissue engineering would generate viable tissue engineered autologous small intestines and transplanted back into the patient.

## Conclusions

The current treatments for MNGIE are inadequate to resolve the GI manifestations, the most common and fatal complications of the disease. Understanding the pathogenesis of the GI dysmotility in MNGIE is limited by the scarcity of the available tissues of patients or the relevant platforms to model GI dysmotility and its development. This understanding, when well established, would facilitate the clinical application for diagnosis, treatment and monitoring of the outcome of treatment of MNGIE patients. ICC should be fundamental in these applications due to vital function in the physiology of GI motility and demonstrated alterations in MNGIE patients. Deficits in ICC and ICC networks could be a primary event or be triggered by the microenvironment of the GI disease. The imbalanced d-Thd and d-Urd nucleosides is a hallmark of MNGIE and account for alterations of mtDNA, including mtDNA depletion in the small intestine of MNGIE patients [28, 30]. This likely leads to mitochondrial failure and cell death. ICC are rich in mitochondria, therefore are likely to be the target cell types affected mostly. For that, deficits of ICC could be viewed as the primary event preceding the muscular and neurogenic changes that occur in the MNGIE intestine [31]. Other potential factors contributing to deficits of ICC could be inspired by other metabolic and GI dysmotility disorders. Signaling pathways critically involved in development and maintenance of ICC or ion channels and key regulators of the pacemaker activity of the ICC or the intrinsic quality of ICC could be negatively affected by the disease microenvironment. Altogether, these potential pathological mechanisms, however, require thorough investigation in the context of MNGIE as well as their validation as targets for prevention and therapeutic strategies. Future prospects for GI dysmotility intervention could involve strategies to augment SCF/c-Kit signaling, targeting VSICs, cellular transplantation of ICC cells or MSCs supported by ex vivo or genetic modifications to enhance their homing, engraftment, proliferation and function in the injured intestines. Finally, the technological breakthrough, human-derived intestinal organoids are being employed to further our understanding of GI disease pathophysiology in physiologically relevant settings, and are expected to significantly contribute to personalized medicine, which ultimately would benefit MNGIE patients.

## Abbreviations

BMT: Bone marrow transplantation
CIPO: Chronic intestinal pseudo-obstruction
dNTPs: deoxyribonucleoside triphosphates
dThd: thymidine
dUrd: deoxyuridine
GI: Gastrointestinal
HIOs: Human intestinal organoids
hIPSC: human induced pluripotent stem cells
hPSCs: human pluripotent stem cells
HSCGT: Hematopoietic stem cell gene therapy
HSCT: Hematopoietic stem cell transplantation
ICC: Interstitial cells of Cajal
ICC_MY_: Interstitial cells in the myenteric plexus
LV: Lentivirus
MNGIE: Mitochondrial neurogastrointestinal encephalomyopathy
MSCs: Mesenchymal stem cells
mtDNA: mitochondrial DNA
SCF: Stem cell factor
SMCs: Small muscle cells
VSICs: Voltage-sensitive ion channels
